# Metabolic Predictors in Risk Stratification for Oral Cavity and Oropharyngeal Cancer Patients Undergoing Free Flap Tissue Transfer: A Retrospective Study

**DOI:** 10.3390/cancers18040698

**Published:** 2026-02-20

**Authors:** Darko Solter, Andro Koren, Luciana Koren, Emili Dragaš, Alan Pegan, Goran Geber, Davor Vagić, Andro Košec

**Affiliations:** 1 Department of Otorhinolaryngology and Head and Neck Surgery, University Hospital Center Sestre Milosrdnice, Vinogradska Cesta 29, 10000 Zagreb, Croatia; darko.solter@kbcsm.hr (D.S.); alan.pegan@kbcsm.hr (A.P.); goran.geber@kbcsm.hr (G.G.); davor.vagic@kbcsm.hr (D.V.); 2 School of Medicine, University of Zagreb, 10000 Zagreb, Croatia; andro.koren@student.mef.hr (A.K.); luciana.koren@student.mef.hr (L.K.); emili.dragas@student.mef.hr (E.D.); 3 School of Dental Medicine, University of Zagreb, 10000 Zagreb, Croatia

**Keywords:** free tissue flaps, oral cancer, oropharyngeal cancer, nutritional status, body mass index, systemic inflammatory response, postoperative complications, microvascular reconstruction

## Abstract

**Background/Objectives**: Oral and oropharyngeal cancers are highly prevalent and associated with a high mortality. The primary treatment modality is surgery with free flap reconstruction becoming the gold standard. Our study aimed to assess the anthropometric and metabolic predictors of the occurrence of postoperative complications after free flap reconstruction, and to present a PRISM (Predictive Reconstructive Index for Soft-tissue Microflaps) scoring system. **Methods**: This retrospective cohort study included 92 patients with advanced oral and oropharyngeal squamous cell carcinoma who underwent primary surgical treatment with microvascular free flap reconstruction between 2020 and 2024. Clinical, surgical, and biochemical parameters (operative and flap ischemia time, BMI; serum albumin, total protein, and calcium) were analyzed in relation to postoperative complications. Associations were assessed using correlation analyses and binary logistic regression. Based on the key predictive variables, the PRISM scoring system was developed to estimate perioperative risk. **Results**: Underweight or overweight patients showed higher complication rates (Kruskal–Wallis, *p* = 0.019). Longer surgeries were associated with a higher incidence of SIRS, which in turn correlated with increased flap ischemia (Man-Whitney, *p* = 0.032 and 0.039). Lower albumin and total protein levels on the second postoperative day were linked to more complications (Kruskal–Wallis, *p* = 0.001 and 0.010), as were lower calcium levels on the first postoperative day (*p* = 0.033). Additionally, longer hospitalization was significantly associated with complication severity (*p* = 0.031). The PRISM score stratified patients into low-, moderate-, and high-risk groups. **Conclusions**: Free flap outcomes in oral and oropharyngeal cancer are influenced by SIRS, nutritional status, and surgical factors. Longer surgeries, flap ischemia, low albumin, protein, calcium, and abnormal BMI increased the complication risk. Careful preoperative assessment, optimization of patient condition, and strategies to minimize operative time and ischemia are essential. The PRISM score may help stratify patients and guide clinical decisions.

## Simple Summary

This study aimed to evaluate the clinical, anthropometric, nutritional, and biochemical parameters that influence the outcome of free flap reconstruction in patients with oral cavity and oropharyngeal carcinomas. A retrospective analysis was performed on patients operated on between 2021 and 2024. The included variables were operative duration, flap ischemia time, presence of systemic inflammatory response syndrome (SIRS), body mass index (BMI), serum albumin, total protein, calcium levels, and length of hospitalization. The findings demonstrated a significant correlation between SIRS, operative duration, and ischemia time. Furthermore, deviations in nutritional and biochemical parameters (albumin, calcium, proteins, BMI) were associated with a higher incidence of complications. A PRISM scoring system was suggested based on the findings. In conclusion, these findings provide a foundation for the development of a predictive model and clinical guidelines to support individualized patient optimization before surgery, aiming to improve outcomes in microvascular head and neck reconstruction.

## 1. Introduction

Oral and oropharyngeal cancers together are one of the ten most common groups of malignant tumors globally, more common in the male population, accounting for over 450,000 new cases per year [1,2]. Most are squamous cell carcinomas [3]. The established risk factors are tobacco use and alcohol consumption, although in oropharyngeal cancers, HPV infection plays a significant role in development, and often occurs in younger males who do not fit in the classic oncology profile with standard risk factors [1,4]. However, the role of obesity and concomitant metabolic disorders is increasingly recognized as a newer but relatively frequent comorbidity in highly developed countries, where more than 60% of the adult population has an elevated body mass index (BMI) [5]. These factors can indirectly affect immune surveillance, promote chronic inflammation, and thus contribute to carcinogenesis, which indicates the necessity of lifestyle modification and correction of nutritional status [5]. The occurrence of these cancers is most often the result of the simultaneous action of genetic, epigenetic, and environmental factors, the interaction of which accumulates over time and causes progressive changes in cellular physiology, arising de novo or from premalignant lesions [6]. Due to the high mortality rates of these cancers, early diagnosis is of high importance, mostly through clinical examination and biopsies, with symptoms such as pain, localized bleeding, and localized modifications of the appearance and consistency of the oral mucosa [7].

The treatment should be individualized, and a multidisciplinary approach is essential [8]. Surgery is the primary curative treatment modality, where achievable, with neck dissection and possible adjuvant radiotherapy or radiochemotherapy [9]. Due to the extensive nature of the surgeries and the effort to preserve function and quality of life, free flap reconstructions are becoming the standard of care, with microvascular reconstructions representing the gold standard in the compensation of complex defects in the head and neck area [9]. Free flaps refer to tissue segments whose blood supply is entirely separated from the donor site and transferred to the recipient site using microsurgical techniques, which represent the gold standard in the compensation of complex defects in the head and neck area [10,11]. The outcome of treatment depends on the stage of the disease, the location of the tumor, and the biological characteristics. In oral cancer, five-year survival in stage I is about 86%, while in stage IV it falls below 40% [12]. For oropharyngeal cancers, survival varies from 59% in the initial stages to 29% in advanced ones, but patients with HPV-positive tumors generally have a more favorable prognosis in all stages of the disease [12]. In addition to these clinical indicators, more attention is paid to laboratory parameters such as the levels of albumin, C-reactive protein, and the presence of a systemic inflammatory response, which could serve as predictors of the success of surgical and reconstructive treatment [13,14].

Complications can occur both in the donor area (hematoma, seroma, infection, limb weakness) and at the flap level, including partial or complete necrosis, thrombosis, infection, and anastomosis failure [15]. The most common are wound dehiscence, infections, necrosis, and fistulas [16]. The laboratory findings are starting to be assessed as prognostic tools for complication occurrence, as in the context of this research, the free flap can be viewed as a dynamic biological model of the interaction between the patient’s systemic status, local microvascular perfusion, and immune modulation [14]. By comparing clinical and laboratory parameters in our patients, the goal is to develop predictive models for assessing the success of reconstruction.

The aim of our study was to assess the anthropometric and metabolic predictors (duration of surgery, duration of flap ischemia, variations in BMI, albumin, total protein, and calcium) on the occurrence of postoperative complications after free-flap reconstruction in patients with oral or oropharyngeal cancer. Based on our analysis, we developed a PRISM (Predictive Reconstructive Index for Soft-tissue Microflaps) scoring system.

## 2. Materials and Methods

The research was approved by the Ethics Committee (Class: 003-06/25-03/036, Reg. No.: 251-29-11/3-25-06), adhering to the Revision of the Helsinki Declaration of 1989, and the written informed consent of all participants was obtained.

This is a retrospective cohort observational study including patients with pathohistologically verified oral and/or oropharyngeal cancer that underwent surgical treatment and concomitant microvascular free flap reconstruction at a tertiary surgical center between January 2020 and December 2024. All patients received adjuvant (chemo)radiotherapy.

The inclusion criteria were: (1) patients aged 18 to 80 years, (2) patients with oral and/or oropharyngeal SCC, (3) advanced stage disease (III, IVa and IVb), (4) no prior oncological or surgical treatment, (5) no inflammatory or hematological disorders affecting inflammatory metabolism and anti-inflammatory responses, (6) complete medical records, and (7) a minimum follow-up period of 3 months, while complications were recorded in the first 14 days following surgery.

The exclusion criteria were: (1) active preoperative inflammatory disease or infection, (2) early stage of the disease, (3) presence of distant metastases, and (4) incomplete medical data and/or monitoring. A total of 92 patients were included in the study, with no losses in follow-up.

To monitor complications, the following variables were analyzed: duration of surgery, duration of flap ischemia, biochemical parameters (BMI, albumin, total protein, calcium) on the first and second postoperative day, duration of hospitalization, and the presence and type of complications, including SIRS (systemic inflammatory response), flap ischemia, and flap survival. The association between the duration of surgery and ischemia and the values of key biochemical parameters with the occurrence of complications was also analyzed. SIRS was defined by the presence of 2 out of the 4 criteria: body temperature >38 °C or <36 °C, heart rate >90 bpm, respiratory rate >20 breaths/minute or PCO_2_ < 32 mmHg, and leukocyte count >12,000/μL, <4000/μL or >10% immature forms.

The variables are grouped into logical sets: age, sex, TNM category and stage of the disease, tumor localization (oropharynx, oral cavity), type of free flap (anterolateral thigh—ALT; radial forearm—RFFF; deep circumflex iliac artery—DCIA; fibular osteocutaneous flap—FOCFF; scapula; latissimus dorsi), inflammatory indices, presence of comorbidities (diabetes mellitus, hypertension, chronic liver disease, heart failure), and data on smoking and alcohol consumption. Data were collected from preoperative and postoperative medical records through uniform administrative forms. The initial point of follow-up was the patient’s arrival in the recovery room, and the final occurrence of complications within 14 days of the postoperative period. Each complication was recorded separately as a binary censored variable. Complications included the occurrence of a fistula, free flap necrosis, delay in the blood supply to the flap, and complications at the donor site or operating area. If the patient had several complications at the same time or consecutively, each was recorded as a separate event. In the analysis, complication status was operationalized as a four-category nominal variable (0 = no complications, 1 = local infection of the postoperative field, 2 = postoperative hematoma and 3 = occurrence of fistula). In addition, the presence of any complication versus none was analyzed using a binary logistic regression model. The variables regarding flap types, complication presence and clinical covariates used in the reconstruction were analyzed with labeling categorical and scale variables during analyses.

### 2.1. Statistical Analysis

The variables tested are described by standard descriptors (arithmetic mean and standard deviation or median). For statistical analysis, the Spearman and Pearson correlation tests were used to assess linear and nonlinear relationships between variables. Binary logistic regression was used to analyze the correlation of various factors with the occurrence of complications. For nonparametric data, Mann–Whitney and Kruskal–Wallis tests were used, depending on the number of groups that were compared. *p*-values ≤ 0.05 were considered statistically significant. All statistical significance tests were performed with a two-sided Type I error level of 5%. Statistical analysis was performed in MedCalc (version 11.2.1 © 1993–2010, MedCalc Software bvba, Broekstraat 52, 9030 Mariakerke, Belgium).

### 2.2. The PRISM Scoring System

The PRISM scoring system was developed as an exploratory and preliminary score combining clinical and laboratory parameters proven to be significant in predicting postoperative complications in patients undergoing microvascular reconstructions. Scoring was based on validated cut-off values obtained by ROC analysis and the clinical severity of each variable. Based on the PRISM scores, we suggested its possible perioperative use.

## 3. Results

The study included 92 patients diagnosed with oral and/or oropharyngeal cancer, who underwent free flap reconstruction during the primary surgical treatment of the disease. The mean age of the patients was 63.25 years, with a range of 30 to 86 years.

The binary logistic regression model has identified several variables to be associated with the presence of complications: lowered total protein levels (OR 4.4, *p* = 0.35), lowered serum calcium levels on the second postoperative day (OR 4.77, *p* = 0.029), increasing length of hospitalization (OR 4, *p* = 0.045), longer duration of surgery (OR 4.5, *p* = 0.034), free flap ischemia (OR 3.83, *p* = 0.05) and using an antero-lateral thigh (ALT) free flap for reconstruction (OR 7.29, *p* = 0.007).

### 3.1. Correlation of BMI and the Incidence of Complications

**Figure 1:**
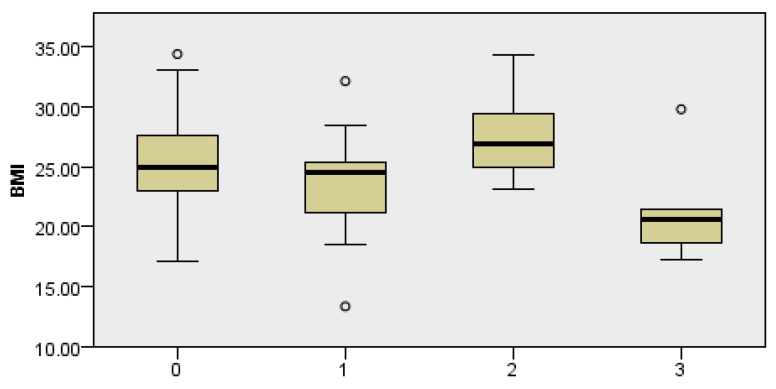
Dependence of postoperative complications in relation to the BMI value. Complications are listed according to their characteristics, with 0 states without complications, 1 state of local infection of the postoperative field, 2 occurrences of postoperative hematoma, and 3 occurrences of fistula.

The body mass index (BMI) of the patients was analyzed, obtaining a test statistical score of 17.056 with two degrees of freedom and an asymptotic significance of 0.002, indicating a statistically significant difference in BMI between the groups. The analysis suggests that patients with a normal BMI have the lowest risk of complications. In contrast, groups with higher and lower BMIs have a significantly higher complication rate (Kruskal–Wallis test, *p* = 0.019) (Figure 1). Complications (as in complications of the operation) are listed according to their characteristics stated above.

### 3.2. Correlation of SIRS and Duration of Surgery

**Figure 2:**
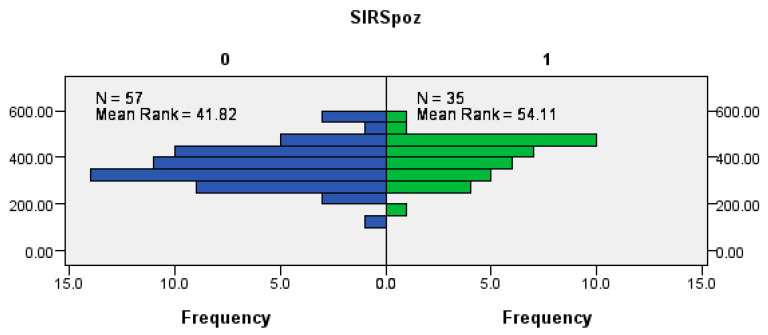
Correlation between SIRS positivity (SIRSpoz) and the duration of the surgery in minutes.

The results suggest a significant correlation between the duration of surgery and the development of SIRS. Patients who underwent longer surgeries were more likely to develop SIRS compared to those with shorter surgery durations (Mann–Whitney U test, *p* = 0.032) (Figure 2).

### 3.3. Correlation of SIRS-Positive Patients and the Incidence of Ischemia

**Figure 3:**
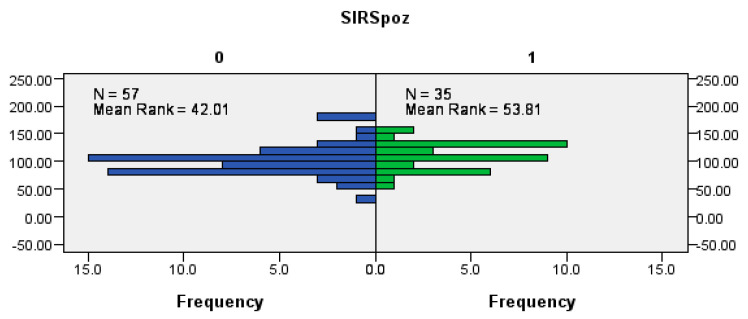
Correlation between SIRS positivity (SIRSpoz) and flap ischemia in minutes.

There is a significant association between SIRS-positive patients (SIRSpoz, Figure 3) and patients with flap ischemia. Patients with SIRSpoz = 1 (those who have SIRS) tend to have higher values of flap ischemia compared to patients with SIRSpoz = 0 (those who do not have SIRS) (Mann–Whitney U test, *p* = 0.039) (Figure 3).

### 3.4. Correlation Between Albumin Levels on the First Postoperative Day and Postoperative Complications

The test did not find a statistically significant association of albumin on the first postoperative day with the incidence of complications. There is no evidence that the groups with complications consistently had a lower median albumin compared to the other groups. Similarly, no trend has been observed to show that higher albumin levels are associated with fewer or more complications.

### 3.5. Correlation of Albumin Levels on the Second Postoperative Day and the Incidence of Complications

**Figure 4:**
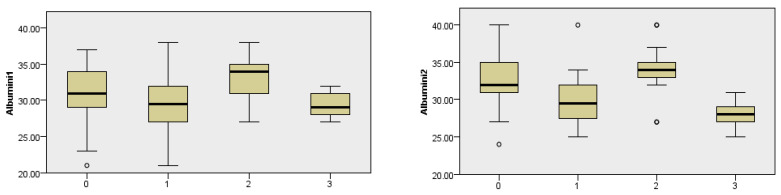
Dependence of postoperative complications in relation to albumin concentration on the 1st postoperative day (Albumini1), albumin concentration on the 2nd postoperative day (Albumini2), and total protein concentration on the 2nd postoperative day (Total Protein 2). Complications are listed according to their characteristics, with 0 states without complications, 1 state of local infection of the postoperative field, 2 occurrences of postoperative hematoma, and 3 occurrences of fistula.

There is a significant association between the levels of albumin on the second postoperative day and the incidence of complications. Higher (or normal) levels of albumin on the second postoperative day are associated with fewer complications. Lower levels of albumin on the second postoperative day are associated with a higher number of complications (Kruskal–Wallis test, *p* = 0.001) (Figure 4).

### 3.6. Correlation of Total Protein Levels on the Second Postoperative Day and the Incidence of Complications

**Figure 5:**
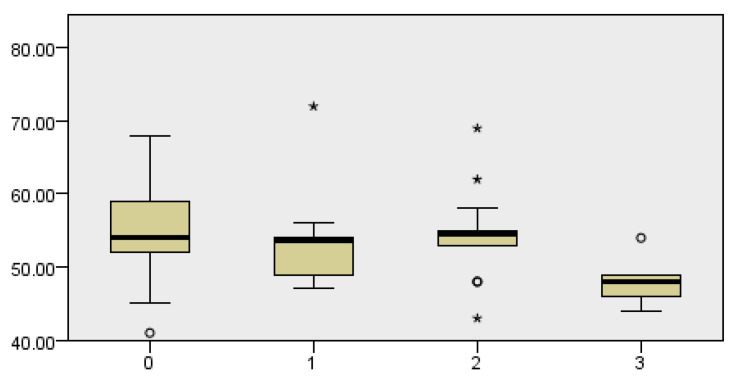
Dependence of postoperative complications in relation to total protein concentration on the 2nd postoperative day (Total Protein 2). Complications are listed according to their characteristics, with 0 states without complications, 1 state of local infection of the postoperative field, 2 occurrences of postoperative hematoma, and 3 occurrences of fistula. The asterix symbol denotes significant differences.

The results of the Kruskal–Wallis test indicate significant differences in total protein levels on the second postoperative day among different groups of the incidence of complications. In particular, as the level of complications increases, protein levels tend to decrease, which may indicate an association between complications and protein levels in the population studied (Kruskal–Wallis test, *p* = 0.010) (Figure 5).

### 3.7. Correlation Between Serum Calcium Levels on the First Postoperative Day and Postoperative Complications

**Figure 6:**
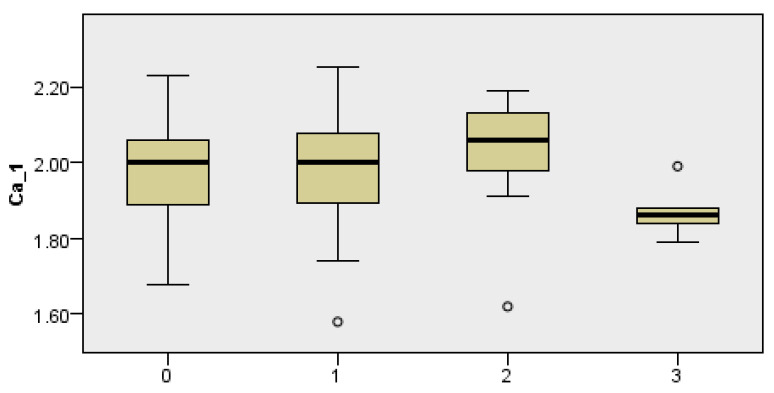
Dependence of the incidence of complications (Complications) in relation to calcium level on the 1st postoperative day (Ca_1). Complications are listed according to their characteristics, with 0 states without complications, 1 state of local infection of the postoperative field, 2 occurrences of postoperative hematoma, and 3 occurrences of fistula.

There is a statistically significant difference in calcium levels (Ca_1) between the different categories of postoperative complications. Lower levels of calcium in certain categories may indicate more serious complications or poorer health outcomes, which may suggest a deficiency or unfavorable physiological response (Kruskal–Wallis test, *p* = 0.033) (Figure 6).

### 3.8. Correlation of the Length of Hospitalization and Complications of Surgery

**Figure 7:**
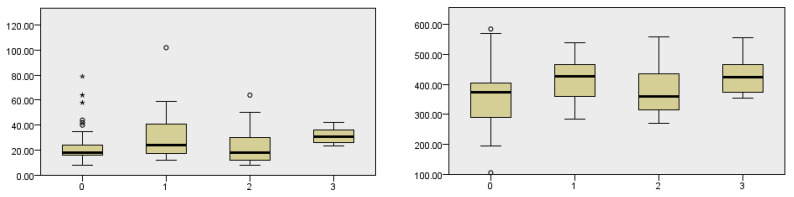
Dependence of the incidence of complications in relation to the time of hospital stay in days and the dependence of complications in relation to the duration of surgery in minutes. Complications are listed according to their characteristics, with 0 states without complications, 1 state of local infection of the postoperative field, 2 occurrences of postoperative hematoma, and 3 occurrences of fistula. The asterix symbol denotes significant differences.

The analysis shows that the length of hospitalizations is significantly related to the severity of complications. As the severity of complications increases (from 0 to 3), the length of hospitalizations also increases, suggesting that patients with more severe complications require a longer hospital stay, which is common in clinical practice. Also, it has been shown that with longer hospitalization, a greater number of complications occur (Kruskal–Wallis test, *p* = 0.004) (Figure 7).

### 3.9. Correlation of the Length of Surgery, Time of Hospital Stay and the Incidence of Postoperative Complications

Surgery length analysis indicates a statistically significant difference in surgery length between groups. The analysis shows that there is a significant correlation between the length of surgery and the severity of complications. Also, the longer the hospital stay, the greater the likelihood of complications (Kruskal–Wallis test, *p* = 0.031) (Figure 7).

### 3.10. The PRISM Scoring System

**Figure 8:**
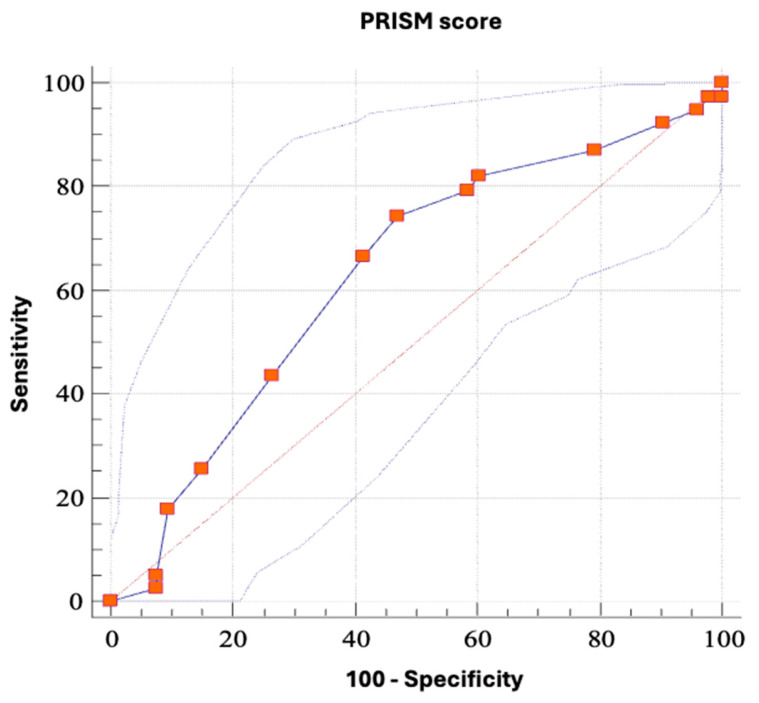
The ROC curve shows the sensitivity and specificity (100-Specificity) of the PRISM model for the occurrence of complications with its 95% confidence interval lines.

The proposed PRISM scoring system for evaluating postoperative complications is presented in Table 1. The sensitivity and specificity of the model are presented in Figure 8.

The included parameters were albumin, BMI, ionized calcium, SIRS, duration of surgery, and time of flap ischemia, parameters shown to be correlated with the occurrence of postoperative complications in our cohort.

The total points of the PRISM models range from 0 to 15. The developed risk categories are as follows:

- Low risk: 0–6 points
- Moderate risk: 7–11 points
- High risk: 12–15 points

**Figure 9:**
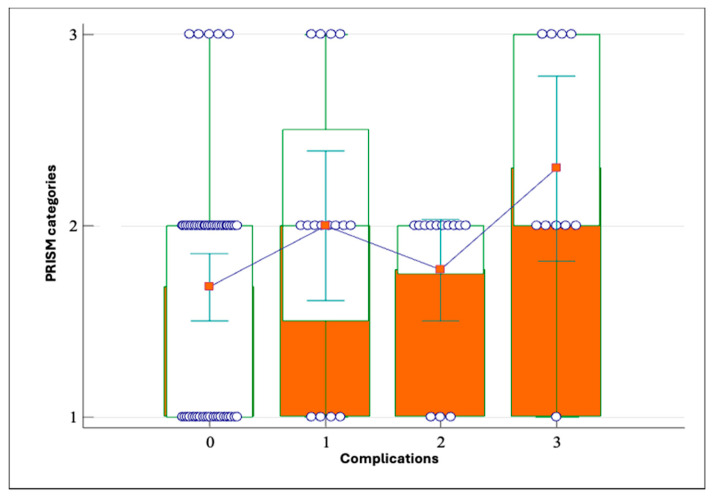
The ratio of PRISM risk categories and the occurrence of complications, where 0—no complications; 1—local infection in the postoperative field; 2—occurrence of hematoma; 3—occurrence of fistula (*p* = 0.039, Kruskal–Wallis test).

The model was tested on a sample of 92 patients, with Youden J being the criterion for the point of highest sensitivity (74.36%) and specificity (52.83%) of the PRISM value of >7 for the occurrence of complications (AUC 0.631, *p* = 0.0274). Then, the predictive value of the limit values and risk categories set in this way was verified. The positive predictive value is 54.3219%, with a 95% CI of 40.226–67.929%, and a negative predictive value of 73.1997%, with a 95% CI of 56.341–86.252%. The relationship between PRISM values and complications was tested, and the results showed that the lower risk category correlates with the severity of complications, in such a way that lower risk categories correlate with the occurrence of local infection and hematoma, and more with the occurrence of postoperative fistulas (*p* = 0.039, Kruskal–Wallis test, Figure 9).

#### The Suggested Use of the PRISM Scoring System

Based on our analysis, we present a possible perioperative use of the developed PRISM scoring system.

Firstly, a risk assessment should be done on the first surgical consultation. Based on the score, patients should be classified into one of the three groups (low, moderate, or high risk).

Secondly, preoperative status should be optimized. In cases of low albumin, high CRP, or altered calcium and magnesium levels, we suggest the following steps.

- Albumin < 35 g/L: Protein diet, enteral supplements
- CRP > 10 mg/L: Postponement of surgery, reevaluation of the inflammatory status
- Correction of Ca^2+^ and Mg, as needed

Thirdly, the intraoperative strategy should be optimized. Our analysis suggests the following:

- Target ischemic time <105 min
- Selection of the flap according to the status of the patient
- Involvement of an experienced team

Finally, in the postoperative surveillance, our analysis suggests the following:

- Clinical follow-up every 2 h for the first 2 days
- Doppler flow control
- Automatic alerts in the EHR if PRISM >7 and vital parameters deviate

## 4. Discussion

The results of our study clearly show that the success of microvascular reconstruction in oncological head and neck surgery is not conditioned solely by the surgical technique but is deeply related to the systemic physiological and molecular conditions of the patient. The identified factors, including hypoalbuminemia, hypocalcemia, BMI, SIRS, ischemia time, and microenvironment dysregulation, seem to form an intertwined network that determines flap perfusion, healing, and survival.

The BMI analysis showed that both higher and lower values were associated with a higher rate of complications, and normal BMI values (18.5–24.9) correlated with the best postoperative outcomes. According to one of the fundamental laws of pathophysiology, the Arndt–Schulz law, all parameters in the body can be observed within the spectrum of homeostasis, and at the end values of the same, the system is exhausted. We can also apply this to our research. In patients with low BMI and nutritional depletion, reduced immune system reactivity is noticeable, leading to a lower capacity for tissue repair and increased susceptibility to infections [17]. Obesity, on the other hand, is now recognized as a chronic inflammatory condition, with adipose tissue now considered an endocrine organ that secretes pro-inflammatory cytokines [18]. This creates a prothrombotic microenvironment that disrupts microcirculation and possibly compromises the vascular anastomosis of the flap [19]. Along with BMI, another nutritional parameter is albumin, with low serum albumin on the second postoperative day being associated with a higher incidence of complications, including infections, flap ischemia, and delayed healing. Similar findings were reported by Tsai et al., who showed that low postoperative albumin levels were associated with wound infections and low preoperative levels were associated with worse survival [20]. According to the ESPEN guidelines (2021), routine nutritional screening is recommended in all patients before major surgical procedures, with timely introduction of nutritional intervention in case of identified risk [21]. Our results support nutritional assessment as an indispensable part of preoperative preparation due to the associations with complications noted in our analysis.

Calcium levels were also analyzed, and patients with hypocalcemia in the early postoperative period had more frequent ischemic flap episodes, delayed healing, and a higher number of vascular complications. Calcium participates as a cofactor in the conversion of prothrombin to thrombin, a key step for the stable formation of the fibrin network, and modulates vascular tone and endothelial function through signaling pathways that include calmodulin, nitric oxide (NO), and protein kinase, which is crucial for the preservation of graft microcirculation [22]. Hence, routine monitoring and timely calcium correction should be part of the perioperative protocol in patients undergoing microvascular reconstructions to possibly preserve flap perfusion, reduce thromboembolic incidents, and improve the overall surgical outcome.

As for the inflammatory response, our results show a strong correlation between the duration of surgery and the incidence of SIRS, which is further associated with flap ischemia and increased postoperative complications. Longer surgeries, especially those exceeding 6 h, were associated with a significantly higher incidence of SIRS, and among SIRS-positive patients, a higher incidence of vascular thrombosis and partial or complete flap loss was also recorded. In the surgical context, SIRS develops as a result of systemic dysregulation of the inflammatory response, whereby protective mechanisms turn into self-destructive ones [23]. This response is influenced by the preoperative period, anesthesia, and the extent of the surgery [23]. Pathophysiologically, the presence of SIRS implies activation of the NF-κB signaling pathway and systemic release of pro-inflammatory cytokines such as TNF-α and IL-6, leading to endothelial dysfunction, microangiopathy, and compromised microcirculation within the flap [24]. These changes directly affect the outcome of microvascular grafting and, in severe cases, could result in complete loss of the flap. Gan et al. in their meta-analysis showed that prolonged duration of surgery in patients with head and neck cancer significantly increased the risk of developing surgical site infections, with each additional time interval of surgery associated with a relative risk increase of 42% [25]. Given that an inflammatory response is inevitable after surgery, the primary objective should be to regulate the duration and severity of the response by reducing operative time, without jeopardizing the final surgical outcome.

Along with the duration of surgery, longer hospitalization was also statistically significantly associated with a higher frequency of postoperative complications. In these patients, infections, flap ischemia, dehiscence, the need for revisions, and prolonged rehabilitation were reported more frequently. Massart et al. showed that hospitalization for more than 14 days doubled the risk of nosocomial infections and, consequently, mortality of patients in intensive care units [26]. Hospital-acquired infections are particularly problematic in microvascular surgery due to the disruption of the delicate balance of the flap microenvironment. In addition, the presence of multidrug-resistant strains (e.g., MRSA, *P. aeruginosa*), as well as prolonged hospitalization with the use of intravenous lines, catheters, and antibiotics, increases the risk of systemic and local complications [27,28,29]. Hospitalization is accompanied by bed rest, which leads to muscle atrophy and insulin resistance even after one week, and consequently, the equilibrium necessary for proper wound healing is disrupted by the fostering of a pro-inflammatory environment at the wound site [30,31]. The occurrence of postoperative complications results in an extended length of hospitalization, demonstrating a dependent relationship between the two variables. Ultimately, the length of hospitalization can be used as a secondary, but valuable clinical indicator of the complexity of the postoperative course. Recognizing factors that contribute to its prolongation (infections, poor nutritional status, hypocalcemia, hypoalbuminemia, SIRS) enables timely intervention and an individualized approach, which can shorten the time of hospitalization and, thus, possibly improve recovery and increase the success of the free flap.

The PRISM score, presented in this study, provides a possible tool for patient stratification by the implementation of the above-stated findings. It is a simple but informative clinical tool that allows early risk assessment of complications in patients after microvascular surgery, especially during the first three postoperative days, when early intervention is more likely to improve outcomes if complications occur. As it is based on our rather small cohort (92 patients), it requires further validation on larger samples, but it creates a basis for further modifications and validation of its use. It can serve as a basis for decisions on the intensity of postoperative surveillance and nutritional support of patients undergoing microvascular surgery.

From a clinical point of view, the recognition of these factors enables the development of personalized perioperative care, in which each patient would be classified according to the risk profile, and interventions aimed at correcting specific parameters: nutritional support in hypoalbuminemia, electrolyte stabilization in hypocalcemia, antioxidant protection in prolonged ischemia, and control of SIRS with the help of pharmacological and metabolic regulation.

The limitations of our study are its retrospective nature and restriction to a single surgical center, followed by a relatively small sample size. There is heterogeneity of the free flap types, which may have influenced outcomes and complication rates. As mentioned, anesthetic and surgical techniques have an impact on postoperative outcomes. These factors were not fully controlled in our study, presenting possible cofounding factors that should be taken into consideration when interpreting the results. The PRISM scoring system is based on a rather small cohort and lacks external validation; hence, further validation of PRISM scores in larger multicenter studies is needed to enable its clinical use.

## 5. Conclusions

This study demonstrates that the outcome of free flap reconstruction in our cohort of oral and oropharyngeal cancer patients depends on a combination of systemic inflammation, nutritional status, and surgical factors. Our findings show that the presence of SIRS, particularly in patients with longer operative and ischemia times, is strongly associated with postoperative complications and flap failure. Poor nutritional and metabolic parameters, including low albumin, protein, and calcium levels, and abnormal BMI, further increase the risk. These results emphasize the importance of a careful preoperative assessment, optimizing the patients’ general condition, and opting for surgical strategies aimed at minimizing operative time and ischemia. Finally, a PRISM scoring system was presented as a tool for patient stratification by the above-stated parameters. Ultimately, these findings can help clinicians make more informed decisions and improve outcomes in microvascular reconstruction.

## Figures and Tables

**Table 1:** An example of a proposed PRISM scoring system for the prediction of postoperative complications in patients undergoing microvascular reconstruction. Source: Own processing (Table S1).

Parameter	Values	Score
Albumin Day 1–2	<30	3
	30–38	2
	38–43	1
BMI	<18.5	3
	18.5–21.5 >30	2 1
Ionized Calcium Day 1	<1.90	2
	1.90–2.00	1
SIRS	Present	3
Duration of Surgery	>540 min 420–540 min	2 1
The Time of Ischemia	>130 min 105–130 min	2 1

## Data Availability

Data is contained within the Supplementary Material.

## Supplementary Materials

The following supporting information can be downloaded at: https://www.mdpi.com/article/10.3390/cancers18040698/s1, Table S1: Original data.

## Abbreviations

The following abbreviations are used in this manuscript:

SIRS	The systemic inflammatory response syndrome
BMI	Body Mass Index
PRISM	Predictive Reconstructive Index for Soft-tissue Microflaps
HPV	Human papillomavirus
SCC	Squamous cell carcinoma
ALT	Anterolateral thigh
RFFF	Radial forearm free flap
DCIA	Deep circumflex iliac artery
FOCFF	Fibular osteocutaneous flap
CRP	C-reactive protein
NO	Nitric oxide
NF-κB	Nuclear factor kappa-light-chain-enhancer of activated B cells
TNF-α	Tumor necrosis factor alpha
IL-6	Interleukin 6
